# A Novel miRNA Y-56 Targeting IGF-1R Mediates the Proliferation of Porcine Skeletal Muscle Satellite Cells Through AKT and ERK Pathways

**DOI:** 10.3389/fvets.2022.754435

**Published:** 2022-03-17

**Authors:** Jie Song, Linlin Hao, Xiangfang Zeng, Rui Yang, Shiyan Qiao, Chunli Wang, Hao Yu, Siyao Wang, Yingying Jiao, Hongyao Jia, Songcai Liu, Ying Zhang

**Affiliations:** ^1^College of Animal Science, Jilin University, Changchun, China; ^2^State Key Laboratory of Animal Nutrition, College of Animal Science and Technology, China Agricultural University, Beijing, China; ^3^Department of Breast Surgery, The First Hospital of Jilin University, Jilin, China

**Keywords:** miRNA, IGF-1R, skeletal muscle satellite cells, proliferation, signaling pathways

## Abstract

As a key regulator of gene transcription and post-transcriptional modification, miRNAs play a wide range of roles in skeletal muscle development. Skeletal muscle satellite cells contribute to postnatal growing muscle fibers. Thus, the goal of this study was to explore the effects of novel miRNA Y-56 on porcine skeletal muscle satellite cells (PSCs). We found that Y-56 was highly expressed in porcine muscle tissues, and its expression was higher in Bama Xiang pigs than in Landrace pigs. The EdU assay, cell counting kit-8, and flow cytometry results showed that Y-56 overexpression suppressed cell proliferation and cell cycle, whereas Y-56 inhibition resulted in the opposite consequences. The results of qRT-PCR and Western blot showed that Y-56 remarkably inhibited the expression levels of cyclin-dependent kinase 4 (CDK4), proliferating cell nuclear antigen (PCNA), and cyclin D1. We identified that IGF-1R was a direct target of Y-56 by dual-luciferase reporter assay. Moreover, IGF-1R overexpression promoted the proliferation and cell cycle process of PSCs and upregulated the expression of CDK4, PCNA, and cyclin D1. Conversely, IGF-1R knockdown had the opposite effect. Furthermore, IGF-1R overexpression partially reversed the inhibition of the cell proliferation and cell cycle process of PSCs and the downregulation of the expression of CDK4, PCNA, and Cyclin D1 caused by Y-56 overexpression. Finally, Y-56 inhibited the protein expression levels of p-AKT and p-ERK. Collectively, our findings suggested that Y-56 represses the proliferation and cell cycle process of PSCs by targeting IGF-1R-mediated AKT and ERK pathways.

## Introduction

Being a domesticated mammal, pig has become an indispensable livestock in people's daily life and is an excellent medical model, because it has remarkable similarities with the human body in body size, anatomy, immunology, physiology, metabolism, dietary habits and evolved similarities (1). Bama Xiang pig (BM), a miniature pig from Guangxi Province, China, is characterized by short body size, slow growth rate, low lean meat rate, and better meat quality and has a skeletal muscle phenotype remarkably different from Landrace pig (LP). LP has a tall body size, fast growth rate, high meat yield, but poor meat quality (2, 3). Elucidating the relevant mechanism of the different formation of skeletal muscles between the two pig breeds will provide a theoretical basis for improving porcine production performance and the utilization of pigs as medical models. Therefore, understanding the mechanism of porcine skeletal muscle growth will be of great importance to elucidate the mechanism of the formation of the differences between BM and LP.

The number of muscle fibers in mammals remains constant after birth; therefore, the growth of skeletal muscle throughout postnatal life is achieved through the hypertrophy of existing muscle fibers (4–6). Studies have shown that the hypertrophy of muscle fibers is related to the increase of DNA content, but myonuclei do not possess the ability to synthesize DNA (7, 8). Satellite cells, as the DNA source of postnatal muscle fiber growth, increase the amount of DNA through proliferation, and then differentiate and fuse with existing muscle fiber to achieve muscle fiber hypertrophy (9). The proliferation of skeletal muscle satellite cells maintains the population of muscle stem cells and provides a large number of muscle-derived cells (10, 11). Therefore, skeletal muscle satellite cells as muscle stem cells play an indispensable role in the growth of skeletal muscles after birth.

MiRNAs are endogenous single-stranded RNA molecules with a length of 20–24 nt that are conserved and non-coding and participate in the regulation of gene expression in organisms. MiRNAs regulate gene expression by targeting the 3′untranslated region (UTR) of genes to inhibit gene translation or directly promote mRNA degradation (12). Many miRNAs are the key regulators of skeletal muscle growth (13, 14). For example, IGF-1 stimulates the upregulation of miR-133 during skeletal muscle formation, which in turn inhibits the expression of insulin-like growth factor type 1 receptor (IGF-1R) and regulates the IGF-1R signaling pathway (15). Previous study has acquired the miRNA and mRNA expression profiles of anterior pituitaries from BM and LP by miRNA microarrays and mRNA-seq (16). Among them, Y-56 is a newly discovered miRNA with high expression in pig, and no relevant studies on Y-56 have been conducted yet.

According to the above analysis, the influences and relevant mechanism of Y-56 on porcine skeletal muscle growth were investigated, which will lay the foundation for understanding the mechanism of the different formations between BM and LP. In the study, the effects of Y-56 on the proliferation of porcine skeletal muscle satellite cells (PSCs) and the relevant mechanism were explored.

## Results

### Y-56 Is Highly Expressed in the Muscle and Upregulated in BM Compared With LP

**Figure 1:**
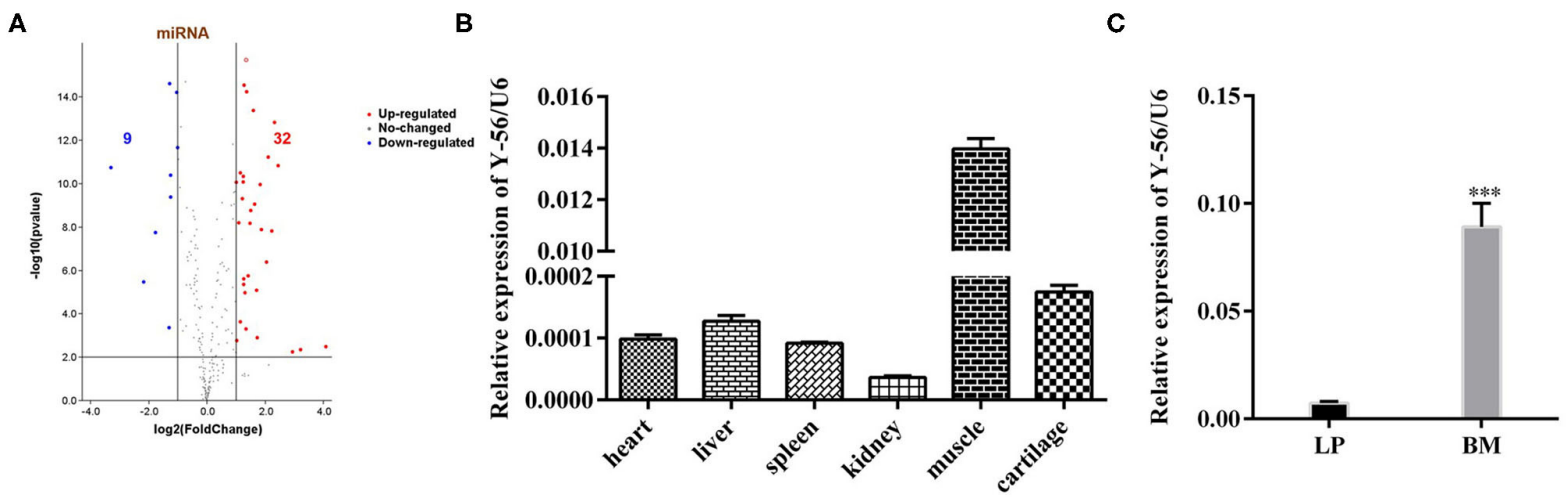
Expression level of Y-56 on the different porcine tissues of LP and BM. **(A)** Volcano plot of the observed fold changes in miRNA expression vs. significance. **(B)** qRT-PCR analysis of Y-56 expression in different porcine tissues. **(C)** MRNA expression level of Y-56 in BM and LP (*P < 0.05, **P < 0.01, and ***P < 0.001).

The miRNA expression profiles were compared in the anterior pituitary between BM and LP. The different expression miRNAs were used in this study. A volcano plot was drawn to show an overview of the differentially expressed miRNAs. The x-axis represents the fold-change of miRNA expression, and the y-axis represents the significance of miRNA expression differences between pools. Differential expression analysis revealed that 41 miRNAs (32 upregulated and 9 downregulated) were regulated significantly in BM after false-discovery rate correction (Figure 1A) (fold change ≥ 1.5, *P* < 0.001).

Among the 32 upregulated miRNAs, there were four novel miRNAs (Y-56, Y-84, Y-71 and Y-44) in the top 10 expression levels in BM (Supplementary Table S1). Furtherly, Y-56 was as a candidate miRNA according to Mean (BM) ≥ 6,000, Log2 (BM/LP) ≥ 1 and *p* ≤ 0.001 (Supplementary Table S2). The expression of Y-56 in other porcine tissues was examined. The results showed that Y-56 was highly expressed in muscle tissues (Figure 1B). Furthermore, the expression levels of Y-56 in leg muscle tissues between LP and BM were compared. The results showed that Y-56 expression in BM was significantly higher than that in LP (Figure 1C; *P* < 0.001).

### Y-56 Suppresses PSC Proliferation

**Figure 2:**
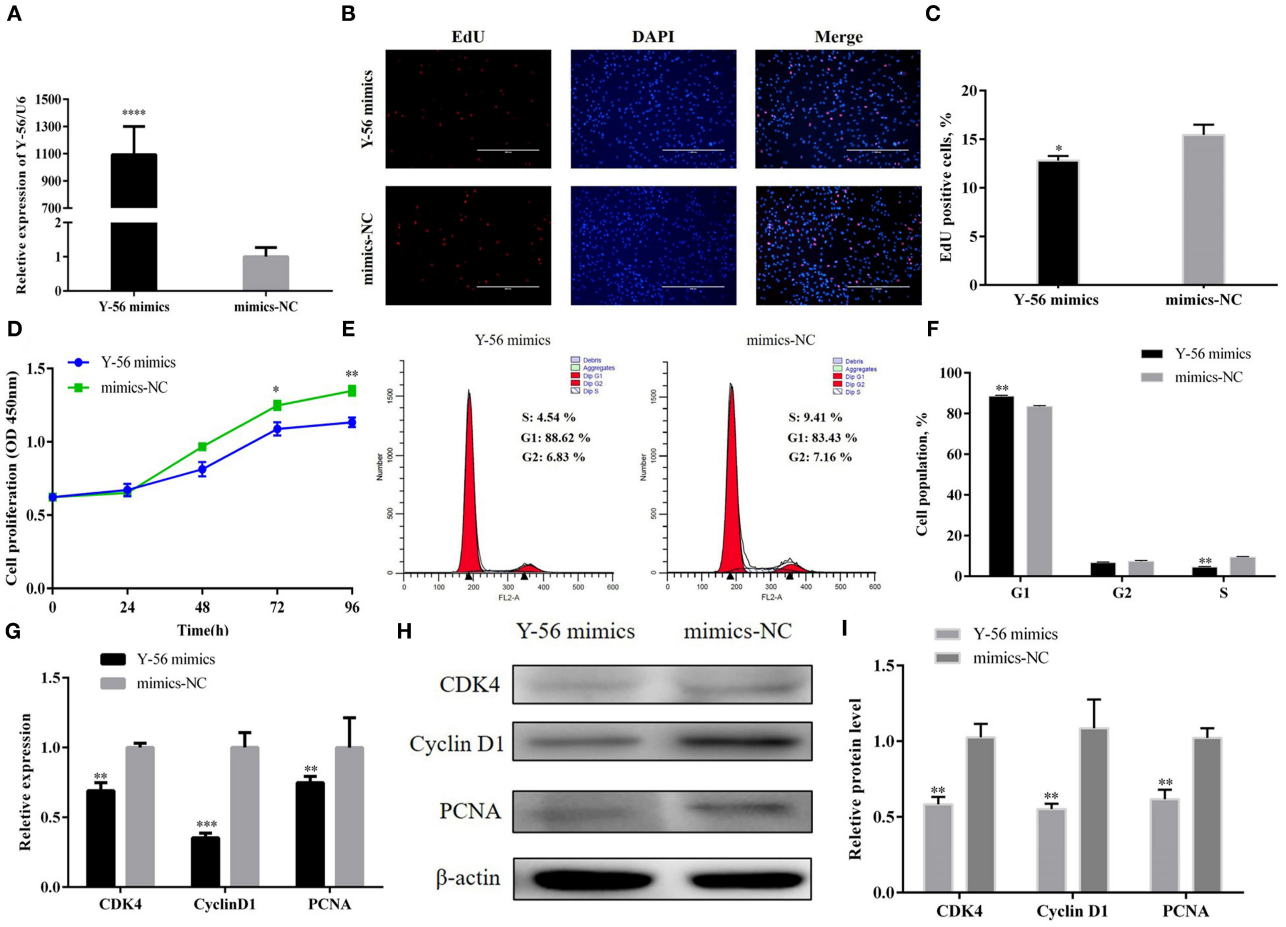
Y-56 overexpression suppressed PSC proliferation. **(A)** Transfection efficiency of Y-56 mimics. **(B)** EdU staining after the transfection of Y-56 mimics in PSCs. **(C)** Statistical results of EdU staining. **(D)** CCK-8 assay after the transfection of Y-56 mimics into PSCs. **(E)** Flow cytometry raw data of the cell cycle analysis of PSCs after Y-56 overexpression. **(F)** Statistical results of cell cycle analysis; **(G)** MRNA expression levels of cell cycle marker genes. **(H,I)** Protein levels of cell cycle marker genes. Results are shown as mean ± SEM, and the data are representative of at least three independent assays. Independent sample *t*-test was used to analyze the statistical differences between groups (*P < 0.05, **P < 0.01, ***P < 0.001, and ****P < 0.0001).

PSCs were isolated according to the above description and then identified by pax7 (Supplementary Figure S1). Y-56 mimics were transfected into the PSCs to reveal the effect of Y-56 on PSC proliferation. The results showed that expression in the Y-56 mimics group highly increased than that in the mimics-NC group (Figure 2A). The 5-ethynyl-2-deoxyuridine (EdU) staining results showed that the number of EdU-labeled positive cells were reduced in the Y-56 mimics group compared with the mimics-NC group (Figures 2B,C; *P* < 0.01). Cell counting kit-8 (CCK-8) assay also showed that Y-56 overexpression hampered PSC proliferation (Figure 2D). In addition, flow cytometry assays showed that the number of PSCs were lower in the G1 phase (*P* < 0.01), whereas the proportion of the PSCs increased in the S phase (Figures 2E,F; *P* < 0.01). When Y-56 was overexpressed, the results of qRT-PCR and Western blot showed that the expression levels of cyclin-dependent kinase 4 (CDK4), proliferating cell nuclear antigen (PCNA), and cyclin D1 substantially decreased (Figures 2G–I).

**Figure 3:**
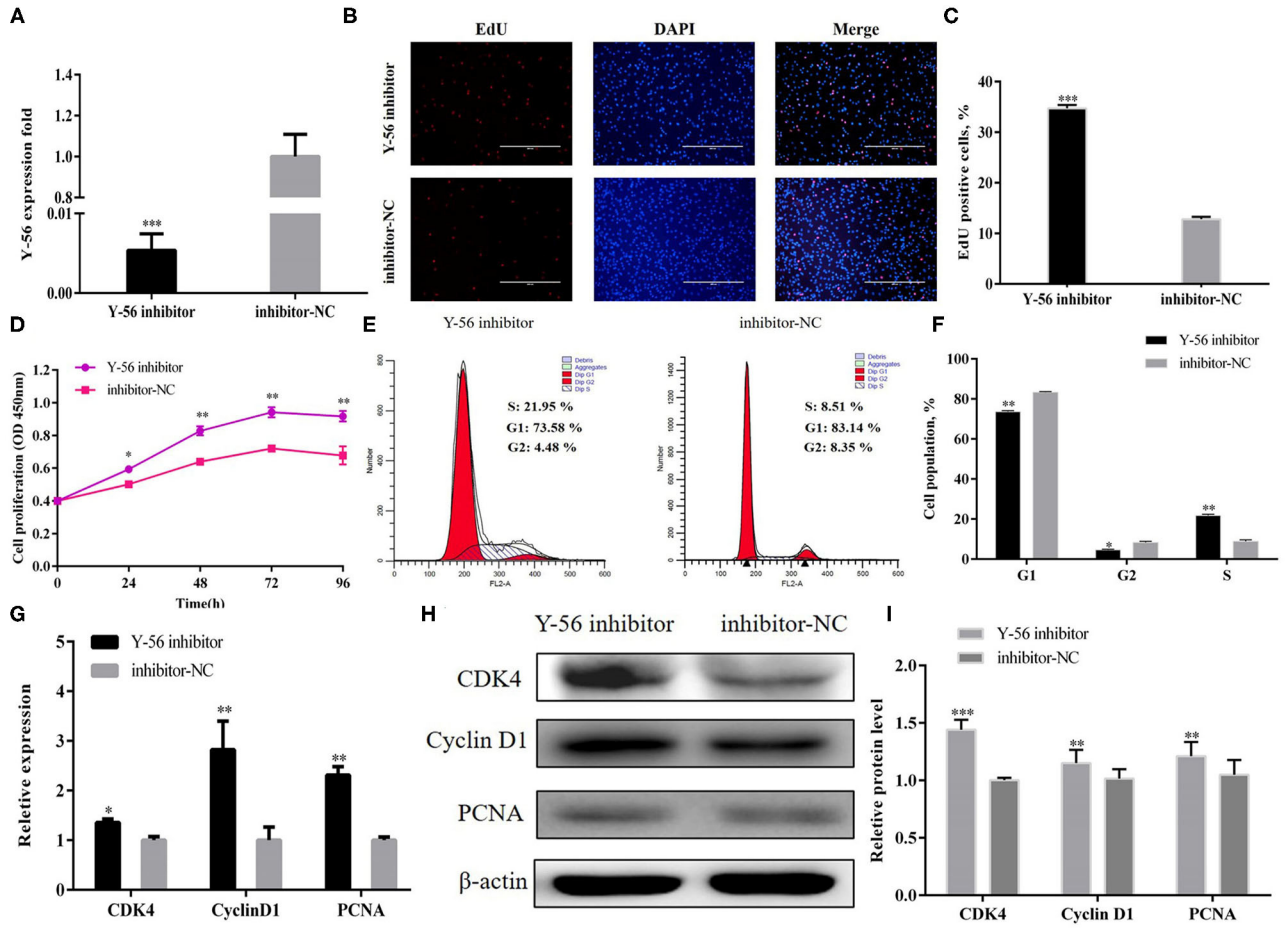
Y-56 inhibition promoted PSC proliferation. **(A)** Transfection efficiency of Y-56 inhibitor. **(B)** EdU staining after the transfection of Y-56 inhibitor in PSCs. **(C)** Statistical results of EdU staining. **(D)** CCK-8 assay after the transfection of Y-56 inhibitor into PSCs. **(E)** Flow cytometry raw data of the cell cycle analysis of PSCs after Y-56 inhibition. **(F)** Statistical results of cell cycle analysis. **(G)** MRNA expression levels of cell cycle marker genes. **(H,I)** Protein levels of cell cycle marker genes. Results are shown as mean ± SEM, and the data are representative of at least three independent assays. Independent sample *t*-test was used to analyze the statistical differences between groups (*P < 0.05; **P < 0.01, and ***P < 0.001).

PSCs were treated with Y-56 inhibitor and inhibitor-NC to further confirm that Y-56 inhibits PSC proliferation. The Y-56 expression in the Y-56 inhibitor group was dramatically reduced (Figure 3A; *P* < 0.001). The number of EdU-positive cells markedly increased in the Y-56 inhibitor group (Figures 3B,C; *P* < 0.001). The number of S-phase cells significantly increased after the suppression of Y-56 expression (Figures 3E,F). qRT-PCR and Western blot data showed that Y-56 inhibition increased the expression of CDK4, PCNA, and cyclin D1 (Figures 3G,I). Therefore, Y-56 overexpression suppressed PSC proliferation, whereas Y-56 knockdown promoted PSC proliferation.

### *IGF-1R* Is a Direct Target Gene of Y-56

**Figure 4:**
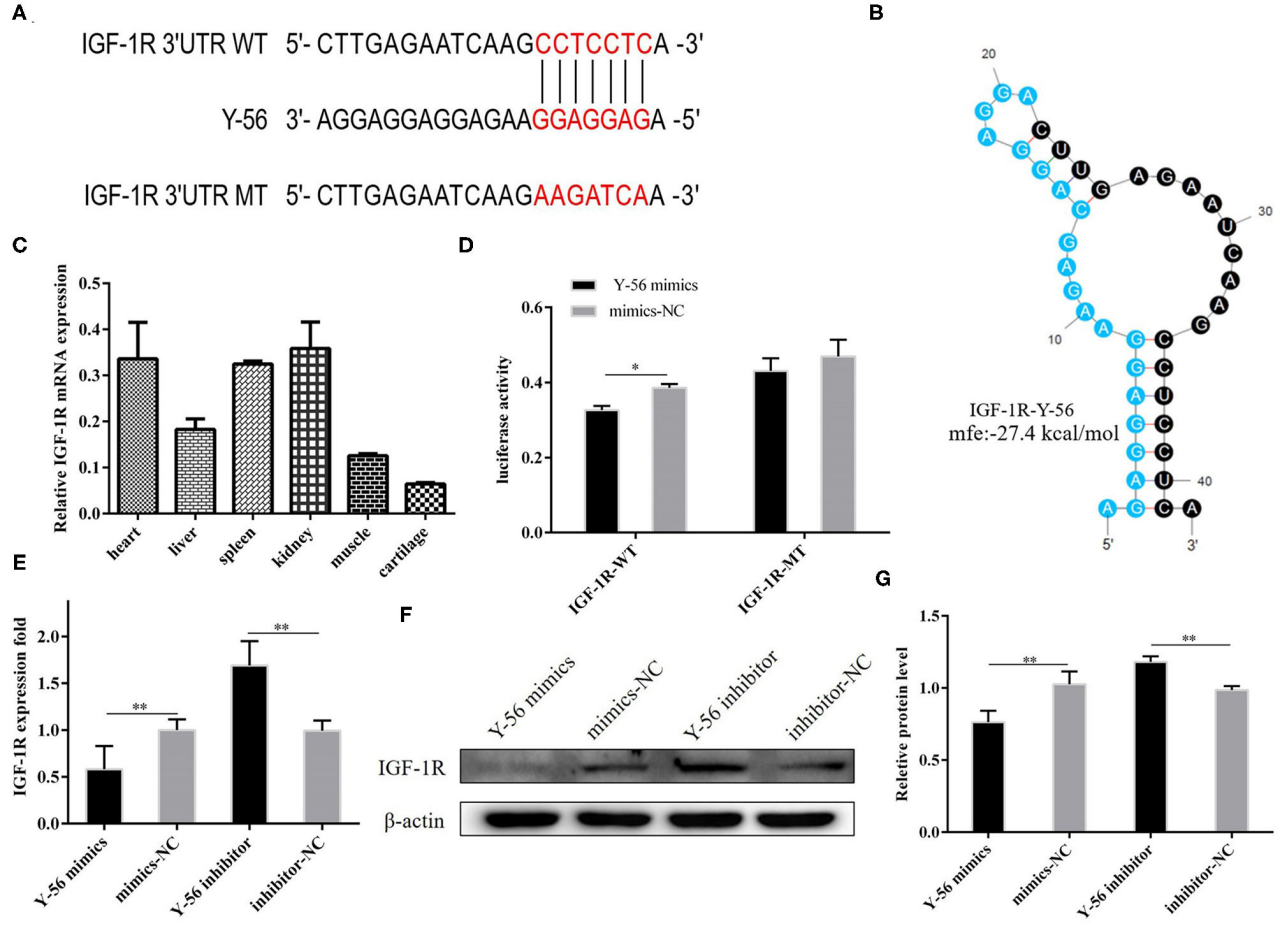
*IGF-1R* is the direct target gene of Y-56. **(A)** Y-56 binding site in the IGF-1R mRNA 3′UTR. **(B)** Potential secondary structures formed by Y-56 binding to IGF-1R mRNA 3′UTR. **(C)** Relative mRNA expression of IGF-1R in different porcine tissues. **(D)** Dual-luciferase reporter assay of the co-transfection of wild-type or mutant IGF-1R 3′UTR with Y-56 mimics or mimics-NC in 293T cells. **(E–G)** MRNA and protein levels of IGF-1R in PSCs after transfection with Y-56 mimics or Y-56 inhibitor (*P < 0.05, **P < 0.01, and ***P < 0.001).

The results in Figures 2, 3 demonstrated that Y-56 inhibited PSC proliferation. TargetScan 7.2 and miRTar Base were used to predict potential target genes to better understand its regulatory mechanisms, and *IGF-1R* was a candidate target gene. The seed sequence of Y-56 is presented in Figure 4A, and the secondary structures formed by Y-56 after binding to the 3′UTR of *IGF-1R* is shown in Figure 4B. The corresponding minimum free energy formed by Y-56 and *IGF-1R* was −27.4 kcal/mol. Then, wild-type *IGF-1R* 3′UTR (IGF-1R-WT) and mutant *IGF-1R* 3′UTR (IGF-1R-MT) dual-luciferase reporter vectors were constructed accordingly. The dual-luciferase activity of IGF-1R-WT and Y-56 mimics co-transfected into 293T cells was higher than that of IGF-1R-WT co-transfected with mimics-NC, and the dual-luciferase activity of IGF-1R-MT with Y-56 mimics and mimics-NC appeared to have no effect (Figure 4D; *P* < 0.01). Next, the mRNA expression levels of IGF-1R in different porcine tissues were detected. The results showed that IGF-1R mRNA expression level was less expressed in the muscle than in other tissues (Figure 4C). Y-56 mimics were transfected into PSCs to verify whether Y-56 regulated the expression of endogenous *IGF-1R*. The results of qRT-PCR and Western blot suggested that IGF-1R mRNA and protein levels were reduced in the Y-56 mimics group than in the mimics-NC group, (Figures 4E–G; *P* < 0.01).

### IGF-1R Promotes PSC Proliferation

**Figure 5:**
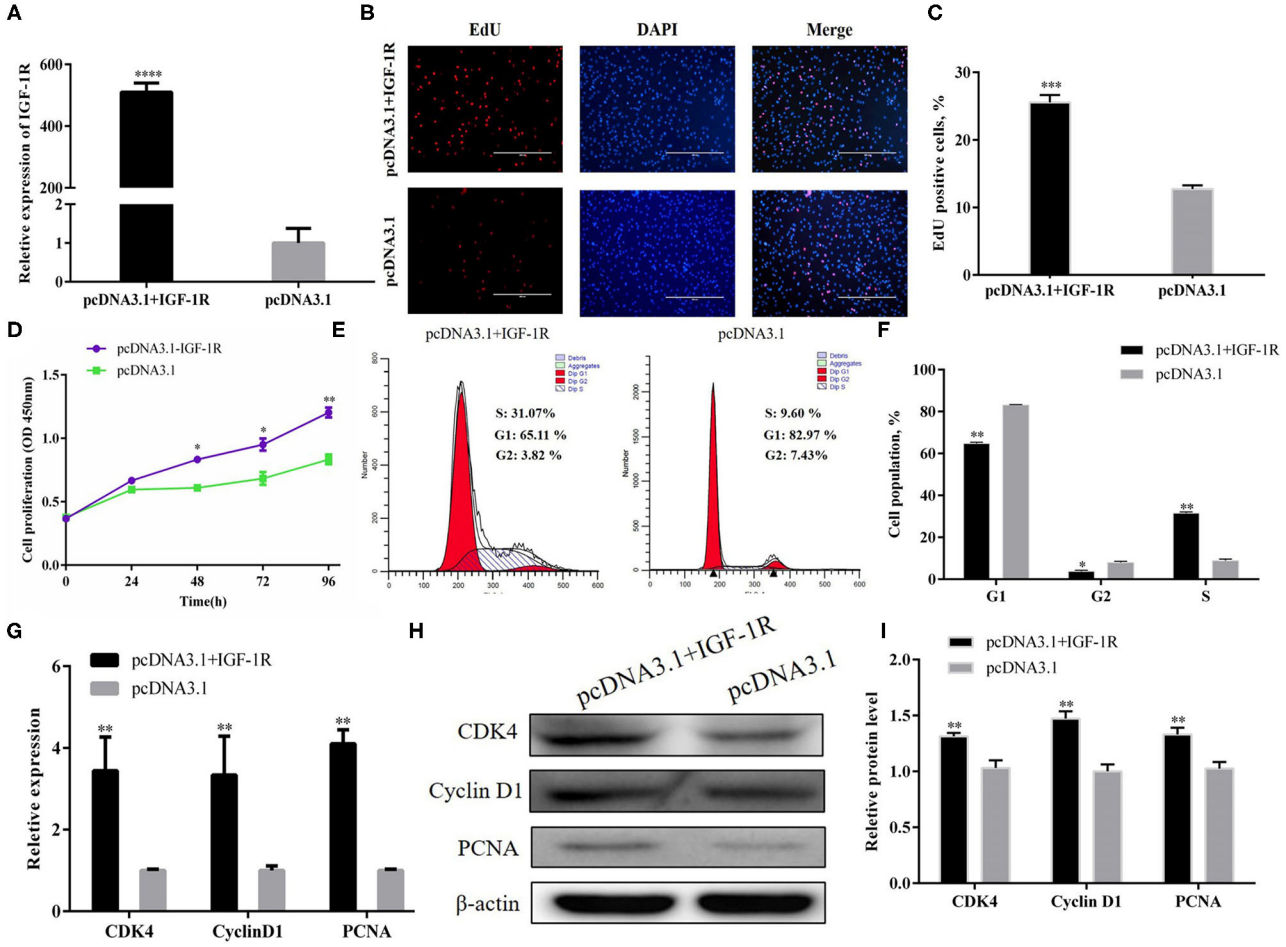
IGF-1R facilitated PSC proliferation. **(A)** Transfection efficiency of IGF-1R overexpression. **(B)** EdU staining after the transfection of IGF-1R overexpression in PSCs. **(C)** Statistical results of EdU staining. **(D)** CCK-8 assay after the transfection of pcDNA3.1+IGF-1R into PSCs. **(E)** Flow cytometry raw data of the cell cycle analysis of PSCs after IGF-1R overexpression. **(F)** Statistical results of cell cycle analysis. **(G)** MRNA expression levels of cell cycle marker genes. **(H,I)** Protein levels of cell cycle marker genes. Results are shown as mean ± SEM, and the data are representative of at least three independent assays. Independent sample *t*-test was used to analyze the statistical differences between groups (*P < 0.05, **P < 0.01, ***P < 0.001, and ****P < 0.0001).

A pcDNA3.1+IGF-1R expression vector was constructed to address the function of IGF-1R in the regulation of PSC proliferation. The mRNA expression of IGF-1R was higher in the pcDNA3.1+IGF-1R group than in the pcDNA3.1 group (Figure 5A; *P* < 0.001). The EdU staining and CCK-8 assays were carried out to evaluate PSC proliferation, and the results showed that IGF-1R overexpression significantly increased the number of cells at the proliferative stage (Figures 5B–D; *P* < 0.001). After IGF-1R overexpression, the number of cells in the G1 and G2 phases were significantly reduced (*P* < 0.01), whereas the number of S-phase cells were increased (Figures 5E,F; *P* < 0.01). Furthermore, the results of qRT-PCR and Western blot showed that IGF-1R overexpression also significantly increased CDK4, PCNA, and cyclin D1 expression at the mRNA and protein expression levels (Figures 5G–I; *P* < 0.01).

**Figure 6:**
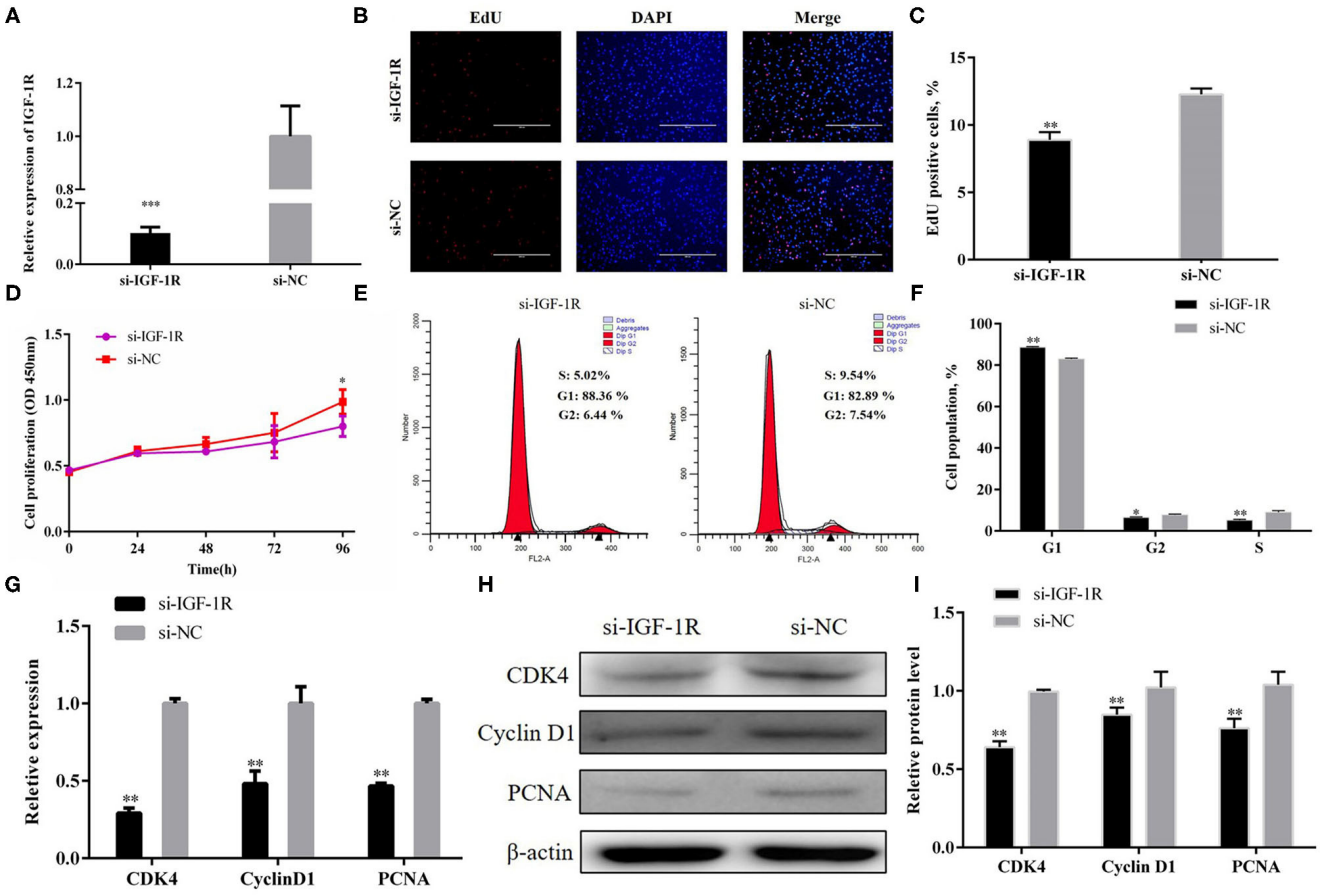
IGF-1R facilitated PSC proliferation. **(A)** Transfection efficiency of si-IGF-1R. **(B)** EdU staining after the transfection of si-IGF-1R in PSCs. **(C)** Statistical results of EdU staining. **(D)** CCK-8 assay after the transfection of si-IGF-1R into PSCs. **(E)** Flow cytometry raw data of the cell cycle analysis of PSCs after IGF-1R knockdown. **(F)** Statistical results of cell cycle analysis. **(G)** MRNA expression levels of cell cycle marker genes. **(H,I)** Protein levels of cell cycle marker genes. Results are shown as mean ± SEM, and the data are representative of at least three independent assays. Independent sample *t*-test was used to analyze the statistical differences between groups (*P < 0.05; **P < 0.01, and ***P < 0.001).

Next, siRNA technology was employed to confirm the role of IGF-1R in PSCs. Si-IGF-1R downregulated IGF-1R expression efficiently at the mRNA level (Figure 6A; *P* < 0.001). EdU staining (Figures 6B,C) and CCK-8 assay (Figure 6D) showed that IGF-1R inhibition hampered PSC proliferation. In addition, the number of G1-phase cells significantly increased after si-IGF-1R transfection, but the number of cells in the S and G2 phases noticeably decreased (Figures 6E,F; *P* < 0.01). Furthermore, when si-IGF-1R was transfected into the PSCs, the expression of CDK4, PCNA, and cyclin D1 were suppressed at the mRNA and protein expression levels (Figures 5G–I; *P* < 0.01). Collectively, the results showed an opposite trend in IGF-1R overexpression, and all proofs demonstrated that IGF-1R could promote PSC proliferation.

### IGF-1R Overexpression Partially Reverses the Inhibitory Effect of Y-56 on PSCs

**Figure 7:**
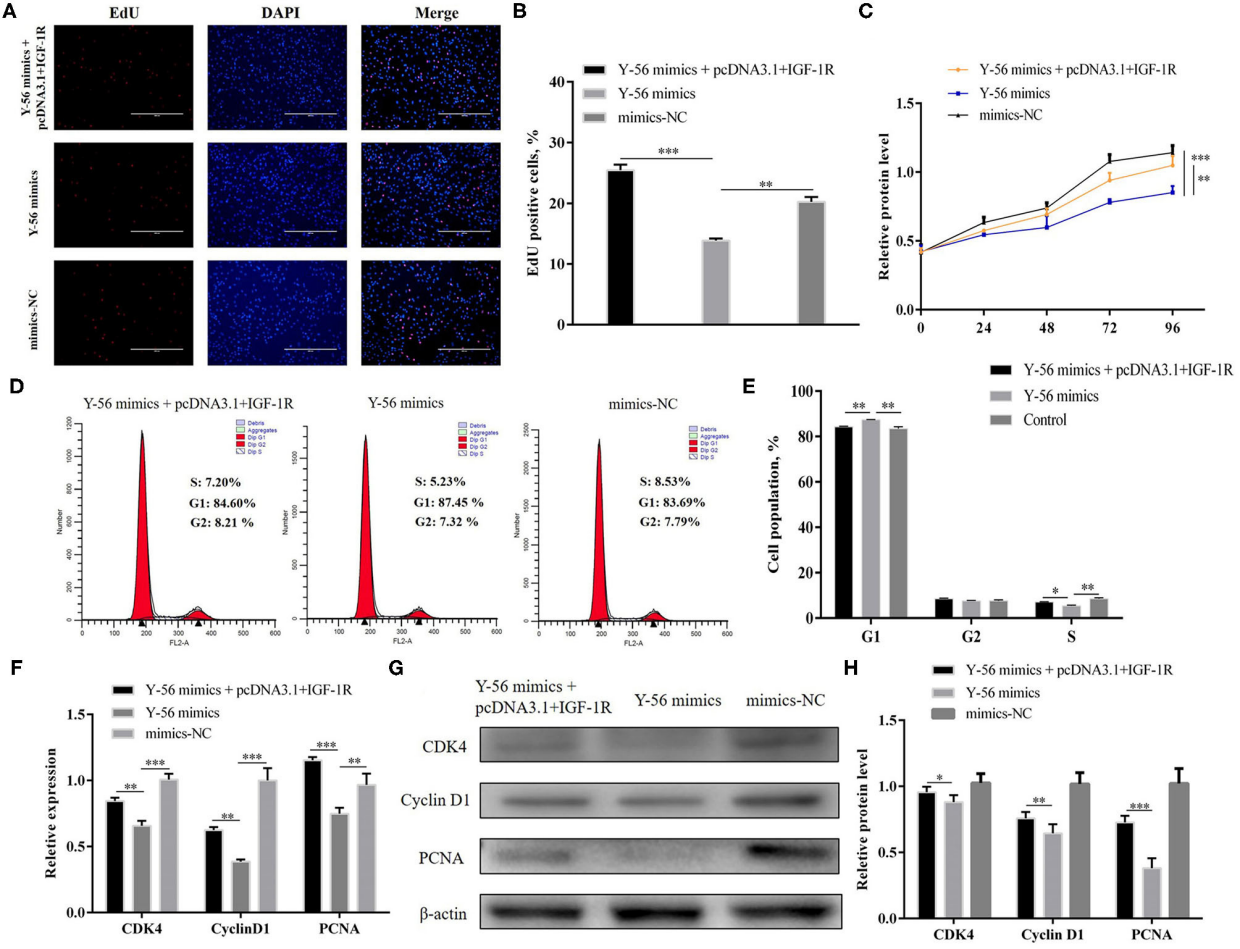
IGF-1R reverses the inhibitory effect of Y-56 on PSCs. **(A,B)** EdU staining assay of PSCs after the co-transfection of IGF-1R and Y-56 mimics. **(C)** CCK-8 assay of PSCs after the co-transfection of IGF-1R and Y-56 mimics. **(D,E)** Flow cytometry data and statistical results of cell cycle analysis after the co-transfection of IGF-1R and Y-56 mimics. **(F–H)**. MRNA and protein expression of cell cycle marker genes after the co-transfection of IGF-1R and Y-56 mimics in PSCs. Results are shown as mean ± SEM, and the data are representative of at least three independent assays. Independent sample *t*-test was used to analyze the statistical differences between groups (*P < 0.05, **P < 0.01, and ***P < 0.001).

pcDNA3.1+IGF-1R and Y-56 mimics were co-transfected to verify whether Y-56 regulated PSC proliferation through IGF-1R. The EdU and CCK-8 assays proved that the decreased proliferation capabilities of PSCs caused by Y-56 overexpression were retarded by IGF-1R overexpression (Figures 7A–C; *P* < 0.01). Moreover, the cell cycle changes also showed that IGF-1R overexpression reversed the inhibition by Y-56 mimics (Figures 7D,E; *P* < 0.01), and the mRNA and protein expression levels of CDK4, PCNA, and cyclin D1 showed the same trend (Figures 7F–H; *P* < 0.001). Collectively, our data suggest that the suppressing role of Y-56 in maintaining PSC proliferation was dependent on IGF-1R.

### Y-56 Inhibits IGF-1R-Mediated AKT and ERK Signaling Pathways

**Figure 8:**
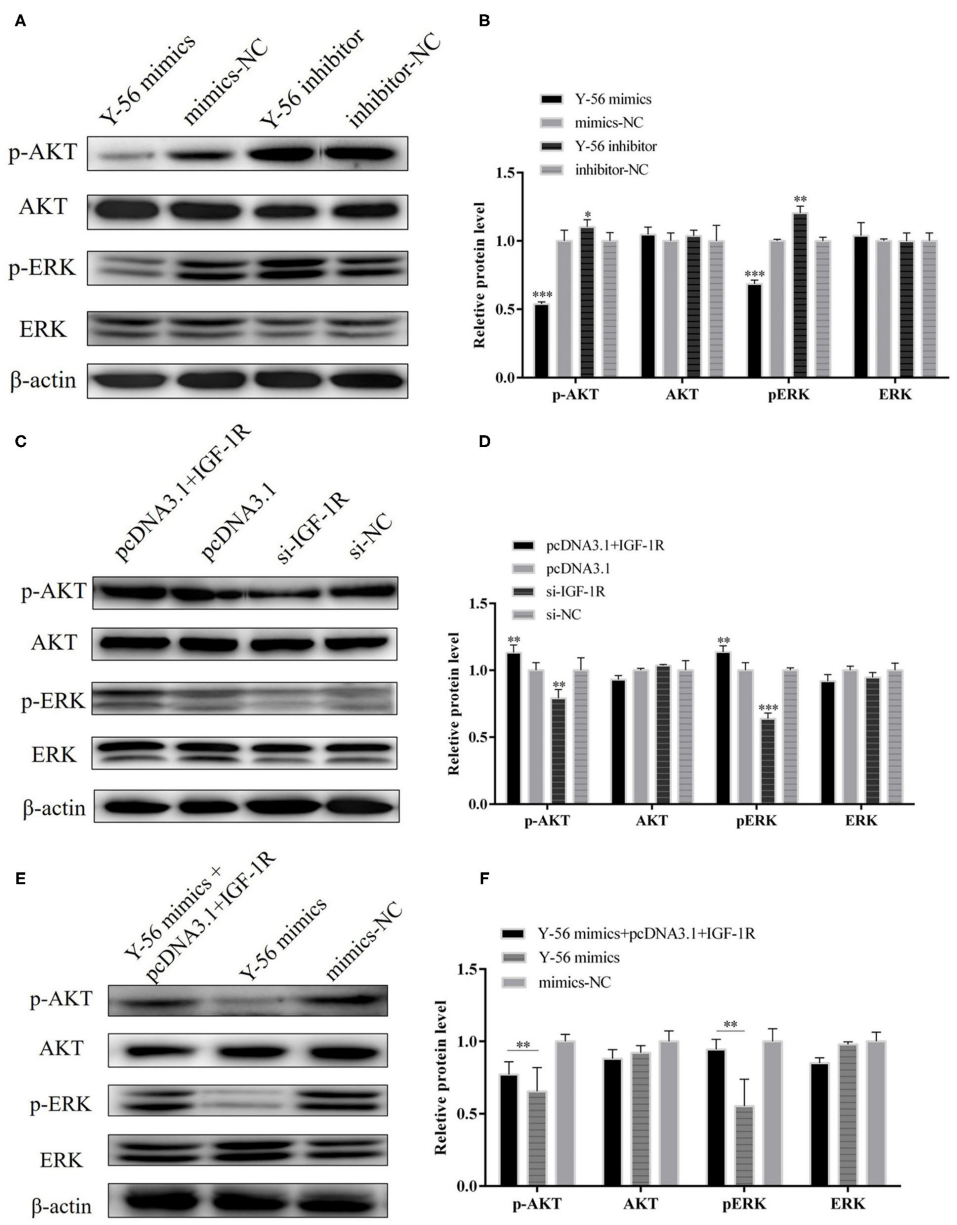
Y-56 inhibited AKT and ERK signaling pathways in PSCs. **(A,B)** The protein expression of p-AKT, AKT, p-ERK, ERK after transfection of Y-56 mimics and Y-56 inhibitor in the PSCs. **(C,D)** The protein expression of p-AKT, AKT, p-ERK, ERK after transfection of 1GF-1R and si-1GF-1R in the PSCs. **(E,F)** The protein expression of p-AKT, AKT, p-ERK, ERK after co-transfection of IGF-1R and Y-56 mimics in the PSCs. Results are shown as mean ± SEM, and the data are representative of at least three independent assays. Independent sample *t*-test was used to analyze the statistical differences between groups (*P < 0.05, **P < 0.01, and ***P < 0.001).

Previous studies reported that IGF-1R activation could trigger the AKT and ERK pathways. Therefore, we examined the possibility that Y-56 restrains the AKT and EKR pathways by targeting IGF-1R. Protein assay showed that Y-56 overexpression remarkably reduced the phosphorylation levels of AKT and ERK without changing the total AKT and ERK expression in PSCs, whereas Y-56 inhibition promoted p-AKT and p-ERK expression (Figures 8A,B; *P* < 0.01). In addition, IGF-1R or si-IGF-1R was overexpressed in PSCs, and the opposite results were obtained (Figures 8C,D). The protein expression levels of p-AKT and p-ERK were recovered in Y-56 mimics-transfected PSCs after transfection with pcDNA3.1+IGF-1R (Figures 8E,F; *P* < 0.01). The results suggested that Y-56 exerts a negative regulatory role in PSCs by directly targeting IGF-1R and triggers the AKT and ERK signaling pathways.

## Discussion

MiRNAs are key factors that regulate the growth and development of skeletal muscles (17). In the prenatal skeletal muscles of LP, Tongcheng, and Wuzhishan pigs, miRNA-133b and miRNA-206 had different expression patterns, which indicates that the same miRNAs play regulatory mechanisms in pigs with different genotypes (18). Skeletal muscle satellite cells bear on the formation of muscle in postpartum animals; they go through cell proliferation, differentiation, and fusion to form muscle tissues (19). Therefore, its proliferation ability will directly affect its differentiation and fusion. This reason is why this paper focused on exploring the effect of miRNA Y-56 on the proliferation of skeletal muscle satellite cells during porcine development.

Many miRNAs are involved in the regulation of skeletal myogenesis. For example, miR-34c inhibits PSCs proliferation but promotes cell differentiation by targeting *Notch1* (20); miR-199b has an inhibitory effect on the proliferation of muscle satellite cells in Large White pigs by targeting the *JAG1* gene (21); and miR-27b promotes muscle development by inhibiting the expression of the *MDFI* gene (22). Similar to previous reports, our EdU staining and CCK-8 assays showed that the novel miRNA, Y-56, suppresses PSCs proliferation by targeting the *IGF-1R* gene (Figures 2–4). However, the effect of Y-56 on the differentiation ability of skeletal muscle satellite cells needs to be further explored.

PSCs proliferation is dependent on an active cell cycle (21). The results of flow cytometry showed that high Y-56 expression also increased the percentage of G1-phase cells and reduced the number of S-phase cells by inhibiting IGF-1R expression (Figures 2E, 5E). In the cell cycle, CDK4 is a kinase that regulates the transition from the G1 phase to the S phase of the cell cycle (23); PCNA is a key factor in DNA replication and cell cycle regulation (24); and cyclin D1 serves as an active switch in the regulation of continued cell cycle progression (25). Consistently, the mRNA and protein expression levels of CDK4, PCNA, and cyclin D1 in PSCs were downregulated by Y-56 overexpression and upregulated by IGF-1R overexpression. Thus, Y-56 inhibits the expression levels of the cell cycle factors, CDK4, PCNA, and cyclin D1, by targeting the *IGF-1R* gene to suppress the cycle process of PSCs. Our results further explored the specific effects of miRNA Y-56 on PSC proliferation from the aspect of cell cycle progression, which will also provide a theoretical basis for the future revelation of the mechanism of miRNA Y-56 in the formation difference between BM and LP.

IGF-1R is an important IGF-1 receptor, and IGF-1 has been proved to have a positive effect on the growth and development of the skeletal muscle (26, 27). IGF-1R is essential for the growth and development of cells and the maintenance of the cell cycle (28–30). Pieces of direct evidence proved that the biological functions of IGF-1R are realized by activating the MAPK/ERK and PI3K/AKT pathways by binding to the ligand IGF-1 (31, 32), which performs vital functions in a wide range of biological processes, including cell proliferation, cell apoptosis, cell migration, and cell invasion (33–35). In our previous study, we found that IGF-1R promotes the proliferation of PK-15 cells, and IGF-1R promotes C2C12 proliferation through the AKT and ERK signaling pathways (36, 37). No such results regarding the function of IGF-1R on PSC has been published previously. However, our results demonstrated that IGF-1R promotes PSC proliferation, and IGF-1R overexpression increased the cell cycle and upregulated the expression levels of marker genes (CDK4, PCNA, and cyclin D1; Figures 5, 6).

**Figure 9:**
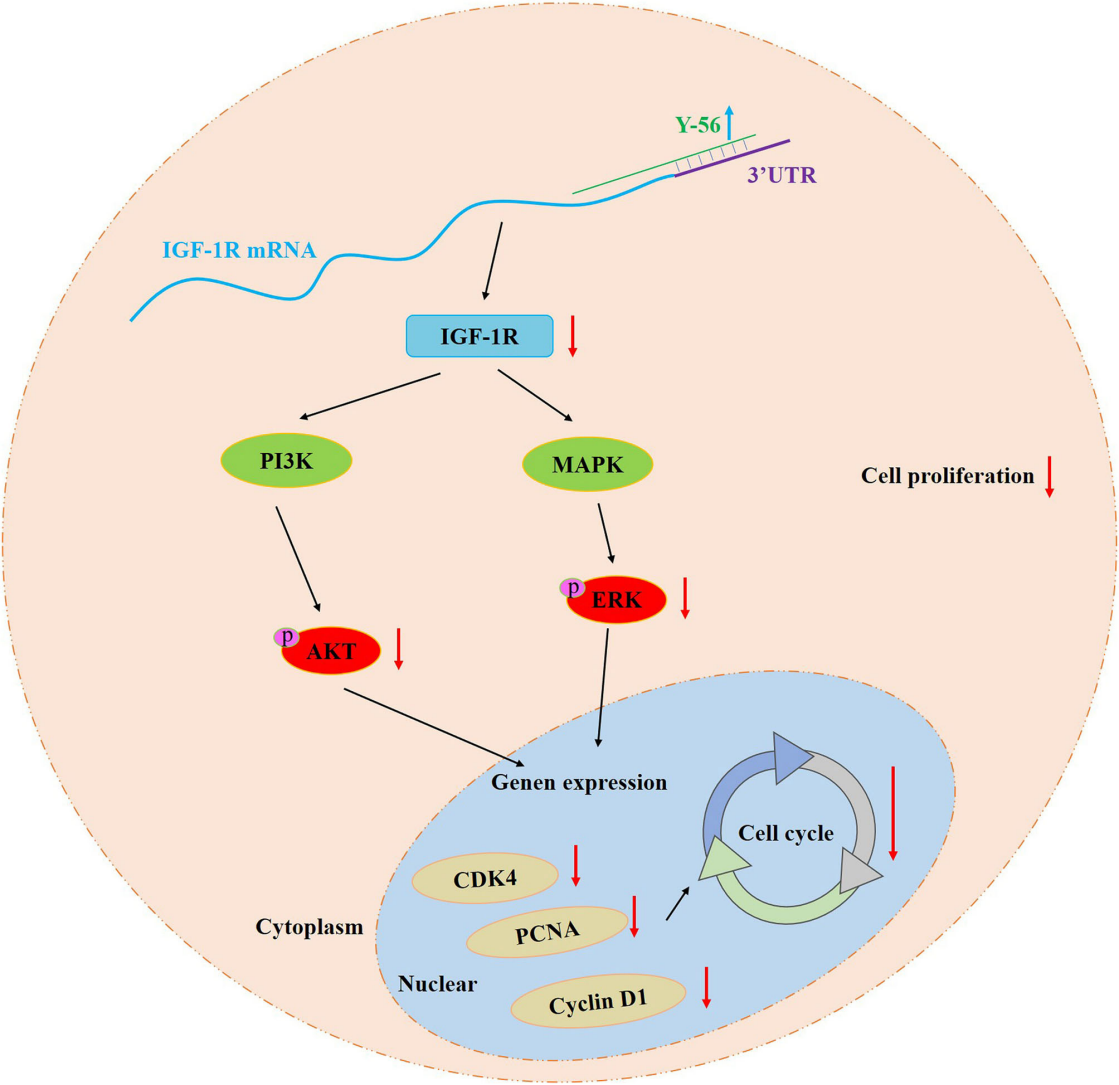
Schematic model of Y-56 regulation on the PSCs proliferation.

Recovery experiment was performed to ascertain that miRNA Y-56 directly targets and inhibits the expression level of IGF-1R and thus affects the proliferation and cycle progression of PSCs. Our results showed that IGF-1R overexpression partially restored the inhibitory effect induced by Y-56 overexpression on cell proliferation and cycle progression. Therefore, the miRNA Y-56–IGF-1R–AKT/ERK pathway was obtained to regulate PSC proliferation (Figure 9). This study provides a new discovery for exploring the function of Y-56 and also provides a theoretical basis for exploring the causes of the differences in skeletal muscle formation between BM and LP in the future.

## Conclusions

Y-56 reduces the mRNA and protein expression levels of IGF-1R by targeting *IGF-1R* mRNA 3′UTR. Y-56 overexpression inhibits the expression of cell cycle-related genes (*CDK4, PCNA*, and *cyclin D1*), as well as the expression of p-AKT and p-ERK, which blocks the cell cycle process and impedes PSC proliferation (Figure 9). In summary, our study provides new insight into the mechanisms of the different formation of the skeletal muscles between BM and LP.

## Materials and Methods

### Animals and Cells

Muscle, heart, liver, spleen, kindey, and cartilage tissues were collected from female BM and LP at 7 days of age, 6 pigs per breed. PSCs were isolated from porcine leg muscle tissues at 7 days of age. Following the method (38). Briefly, the collected leg muscle tissues were washed with PBS supplemented with 0.5% penicillin/streptomycin. Then, the sterile muscle tissues were minced into small pieces and digested with collagenase II (Gibco, USA) at 37°C for 3–6 h. The suspension was filtered through a 70 μm cell strainer and centrifuged at 3000 rpm for 15 min at room temperature. The precipitation was resuspended in PBS, filtered by a 40 μm filter, and centrifuged at 3,000 rpm for 15 min. The supernatant was removed, and the precipitation was resuspended in complete culture medium. The cells were transferred to the culture dish. After 2 h, the suspension was transferred to new culture dish to separate and purify the satellite cells. All animal experiments were performed in accordance with the rules and regulations of the Animal Care and Experimentation Committee of Jilin University (Changchun, China).

### Cell Culture

The 293T cells were cultured in Dulbecco's Modified Eagle Medium (DMEM, Sigma, USA) supplemented with 10% FBS (BI, Israel) and 0.5% penicillin/streptomycin (Gibco, USA) in an incubator with 5% CO_2_ at 37°C. The PSCs were cultured in Dulbecco's Modified Eagle Medium/Nutrient Mixture F-12 (DMEM/F-12, Sigma, USA) with 20% FBS, 0.5% penicillin/streptomycin and 0.5% chick embryo extract (CEE) and were cultured in an incubator with 5% CO_2_ at 37°C.

### Immunofluorescence Staining

PSCs were cultured in 96-well plates, treated with 4% formaldehyde for 15 min, and permeabilized with 0.1% Triton X-100. Next, the cells were blocked in 10% fetal bovine serum (FBS) for 30 min and sequentially incubated overnight with anti-Pax7 (Abcom, US; 1:100). Thereafter, the cells were treated with Alexa Fluor 594-conjugated anti-mouse IgG (Bioworld Technology, USA; 1:200) according to the manufacturer's instructions. Finally, the nuclei were stained with DAPI (Beyotime, Shanghai, China) for 5 min. Cell images were captured with an inverted fluorescence microscope (Nikon, Japan), and the data were analyzed by ImageJ software (National Institutes of Health, Bethesda, MD, USA).

### Plasmid Construction and Cell Transfection

For psiCHECK-2 dual-luciferase reporter vector: The 3′UTR fragment of IGF-1R containing the Y-56 binding site was cloned into the psiCHECK-2 dual-luciferase reporter vector, and the fragments named IGF-1R-WT and IGF-1R-MT were synthesized by Genewiz (Beijing, China). The sequences are listed in Table 1.

**Table 1:** Oligonucleotide sequences used in this study.

**Fragment name**	**Sequence (5′to 3′)**
Y-56 mimics	AGAGGAGGAAGAGGAGGAGGA
	CUCCUCCUCUUCCUCCUCUUU
Mimics-NC	UUCUCCGAACGUGUCACGUTT
	ACGUGACACGUUCGGAGAATT
Y-56 inhibitor	UCCUCCUCCUCUUCCUCCUCU
Inhibitor-NC	CAGUACUUUUGUGUAGUACAA
Si-IGF-1R	CCUCGAGCUAGAGAACUUTT
	AAGUUCUCUAGCUCCGAGGTT

For IGF-1R overexpression vector: The full coding sequence of IGF-1R was synthesized by Genewiz (Beijing, China) and cloned into the pcDNA3.1(+) expression vector.

The RNA oligonucleotides used in this study, including Y-56 mimics, mimics-NC, Y-56 inhibitor, inhibitor-NC, si-IGF-1R, and si-NC, were obtained from Gene Pharma (Shanghai, China) and are shown in Table 1. The oligonucleotides and plasmids were transfected using Lipofectamine 2000 (Invitrogen, USA) according to the manufacturer's protocol with at least three replications.

### Dual-Luciferase Reporter Assay

The psiCHECK-2 dual-luciferase reporter vector (200 ng) containing IGF-1R-WT or IGF-1R-Mut fragment was co-transfected with Y-56 mimics or mimics-NC duplexes (100 nM) into 293T cells in a 96-well plate with six independent repeats. After 48 h of transfection, *firefly* and *Renilla* luciferase activities were measured in a Fluorescence/Multi-Detection Microplate Reader (BioTek, Winooski, VT, USA) using a Dual-GLO Luciferase Assay System Kit (Promega, Madison, WI, USA).

### QRT-PCR

The RNA samples were obtained from the different tissues of 6 pigs each breed. Total RNA was isolated from the collected tissues and PSCs after transfection using RNAiso Plus (Takara, Ostu, Japan). cDNA synthesis for mRNA was carried out using the PrimeScript RT Reagent Kit (Takara, Japan). Reverse transcription reaction for miRNA was performed with the PrimeScript RT Reagent Kit (Takara, Japan) using the specific Bulge-loop miRNA qRT-PCR primers for Y-56 and U6 that were synthesized by Genewiz (Beijing, China). qRT-PCR reactions were carried out using the SYBR Green PCR Master Mix (Thermo Fisher Scientific, MA) according to the manufacturer's instructions on an ABI PRISM 7900HT thermocycler (Applied Biosystems, Foster City, CA, USA). The comparative Ct (2^−Δ*Ct*^) method was used in qRT-PCR data analysis. All amplifications were performed in triplicate, and the mRNA levels of the target genes were normalized using the *GAPDH* gene as the internal reference. The primers used for qRT-PCR are listed in Table 2.

**Table 2:** Primers used in this study.

**Primer name**	**Primer sequences (5^**′**^to 3^**′**^)**	**Size (bp)**
IGF-1R	F: TGCCCTTCAGGCTTCATC	91
	R: TCTTCTCTTCCTCACAGACTTTG	
CDK4	F: TTCGAGCATCCCAATGTTGTC	225
	R: GTCTCGATGAACGATGCAGTTG	
PCNA	CAATTTGGCCATGGGCGTGA	103
	GGTGTCTGCATTATCTTCTGCC	
Cyclin D1	F: TGCATCTACACCGACAACTCCA	222
	R: GTTGGAAATGAACTTCACGTCTGT	
GAPDH	F: TCGGAGTGAACGGATTTGGC	189
	R: TGACAAGCTTCCCGTTCTCC	
U6	F: CTCGCTTCGGCAGCACA	
	R: AACGCTTCACGAATTTGCGT	

### CCK-8 Assay

The cell proliferation rate of PSCs was assessed at 0, 24, 48, 72, and 96 h after transfection by CCK-8 (Beyotime, China). Briefly, the PSCs were seeded in a 96-well format at a density of 5000 cells/well and then transfected with different miRNAs, vectors, or siRNAs as described above. CCK-8 solution (10 μL) was then added to each well containing 100 μL of the culture medium, and then the plate was incubated for 30 min at 37°C. The absorbance was read at a wavelength of 450 nm using a microplate reader (Tecan, Switzerland).

### EdU Staining

The PSCs were seeded in 96-well plates at a concentration of 2 ×10^3^ per well. Then, the PSCs were treated with Y-56 mimics, Y-56 inhibitor, pcDNA3.1+IGF1R, and si-IGF-1R for 48 h and incubated with 50 μM EdU (RiboBio, Guangzhou, China) for 3 h. The cells were washed twice with PBS, fixed with 4% paraformaldehyde for 15 min, and permeabilized with 0.5% Triton-X 100 for 20 min. At the end of each step, the cells were washed twice with PBS for 5 min. According to the kit, the cells were incubated in a mixture of reagents for 30 min. The nuclei were stained with DAPI for 5 min. The stained cells were finally observed on an inverted fluorescence microscope (Nikon, Japan), and the data were analyzed in ImageJ (National Institutes of Health, Bethesda, MD, USA).

### Flow Cytometry

The PSCs were cultured in a 6-well culture plate at a density of 4 × 10^5^ per well and treated with Y-56 mimics, Y-56 inhibitor, pcDNA3.1+IGF1R, and si-IGF-1R for 48 h. Then, the cells were digested with 0.25% trypsin and terminated with Dulbecco's modified Eagle medium/Nutrient Mixture F-12 containing 20% FBS. The cells were collected and fixed in cold 70% ethanol overnight at 4°C. The cells were washed twice and stained with 50 mg/mL propidium iodide (PI) for 30 min. Finally, the cell cycle was analyzed by flow cytometry (Becton Dickinson, USA).

### Western Blot Analysis

The PSCs were harvested after 48 h of transfection to extract proteins using a lysis buffer (KeyGen Biotech, China) complemented with protease and protein phosphatase inhibitor on ice. Protein concentration was determined using Bicinchoninic Acid Protein Assay Kit (Beyotime, China), and 30 μg protein per sample was loaded and separated using 5% stacking gel and 12% separating gel. The separated proteins were transferred to polyvinylidene difluoride membrane, which was then blocked in 5% bovine serum albumin for 2 h at room temperature and incubated with primary antibodies at 4°C overnight. The membranes were washed thrice (10 min each) in tris-buffered saline with Tween and incubated with horseradish peroxidase (HRP)-conjugated goat anti-rabbit IgG or HRP-conjugated goat anti-mouse IgG (Bioworld Technology, USA, 1:2,000) for 2 h at room temperature.

The Western blots were visualized by Enhanced Chemiluminescence (Bioworld, USA) performed on a Bio-Rad Gel Doc XR instrument (Bio-Rad, Hercules, CA, United States). Thereafter, the gray value of the target strip was analyzed using Geno Sens Analysis software, and the expression of the target proteins was analyzed by the ratio of the gray value of the target strip to the gray value of β-actin.

The primary antibodies used in this study were as follows: IGF-1R rabbit polyclonal antibody (Abcom, US, 1:1000), PCNA rabbit polyclonal antibody, CDK4 rabbit polyclonal antibody, cyclin D1 rabbit polyclonal antibody (Cell Signaling Technology, USA, 1:2,000), p-AKT rabbit polyclonal antibody, AKT rabbit polyclonal antibody, p-ERK rabbit polyclonal antibody, ERK rabbit polyclonal antibody, and β-actin polyclonal antibody (BBI, China, 1:2,000).

### Statistical Analysis

All experimental results are presented as the mean ± standard error of the mean (SEM) of at least three independent replications. The statistically significant difference between groups was tested by one-way ANOVA using GraphPad Prism version 6.01. *P* < 0.05 was considered statistically significant (^*^P < 0.05, ^**^P < 0.01, and ^***^P < 0.001).

## Data Availability Statement

The original contributions presented in the study are included in the article/Supplementary Material, further inquiries can be directed to the corresponding authors.

## Ethics Statement

The animal study was reviewed and approved by Institutional Animal Care and Use Committee of Jilin University, IACUC (SY201912002).

## Author Contributions

JS, LLH, SCL, and YZ conceived and designed the experiments. JS and RY performed the experiments. JS, XFZ, CLW, and HY analyzed the data. SYQ, SYW, YYJ, and HYJ contributed to the sample collections and helped to facilitate the experimental process. JS wrote the manuscript. LLH and SCL modified the manuscript. YZ responded to the reviewers' revisions and made changes to the article. JS was responsible for contacting institutions to revise the language quality of the manuscript. All authors have read and approved the final manuscript.

## Funding

This work was supported by the National Natural Science Foundation of China (31772699) and the National Key Research and Development Program of China (2016YFD0500401).

## Conflict of Interest

The authors declare that the research was conducted in the absence of any commercial or financial relationships that could be construed as a potential conflict of interest.

## Publisher's Note

All claims expressed in this article are solely those of the authors and do not necessarily represent those of their affiliated organizations, or those of the publisher, the editors and the reviewers. Any product that may be evaluated in this article, or claim that may be made by its manufacturer, is not guaranteed or endorsed by the publisher.
