# Supplementary material for: A Novel miRNA Y-56 Targeting IGF-1R Mediates the Proliferation of Porcine Skeletal Muscle Satellite Cells Through AKT and ERK Pathways
Source: Front Vet Sci. 2022 Mar 17;9:754435. doi: 10.3389/fvets.2022.754435 (PMC8968951; doi:10.3389/fvets.2022.754435)
Supplement: Supplementary file 1 [file Data_Sheet_1.docx]

**Supplementary file**

**Table S1**

Table S1 The top 10 dysregulated miRNAs based on their expression in BM

| Rank | miRNA | Mean (BM) |
| --- | --- | --- |
| 1 | ssc-let-7a | 14,057 |
| 2 | ssc-let-7f | 13,654 |
| 3 | ssc-miR-30b-5p | 11,402 |
| 4 | ssc-let-7c | 10,659 |
| 5 | ssc-let-7e | 9,231 |
| 6 | ssc-miR-30a-5p | 7,566 |
| 7 | Y-84 | 6,655 |
| 8 | Y-56 | 6,159 |
| 9 | Y-71 | 6,152 |
| 10 | Y-44 | 5,890 |

**Table S2**

Table S2 Different expression of four novel miRNAs between BM and LP

| MiRNA | Log2 (BM/LP) | p-value | Mean (BM) | StDev (BM) |
| --- | --- | --- | --- | --- |
| Y-84 | 0.96 | 2.32E-10 | 6,655 | 425 |
| Y-56 | 1.21 | 4.92E-10 | 6,159 | 406 |
| Y-71 | 0.74 | 8.55E-07 | 6,152 | 560 |
| Y-44 | 1.24 | 8.37E-11 | 5,890 | 352 |

**Figure S1**

**
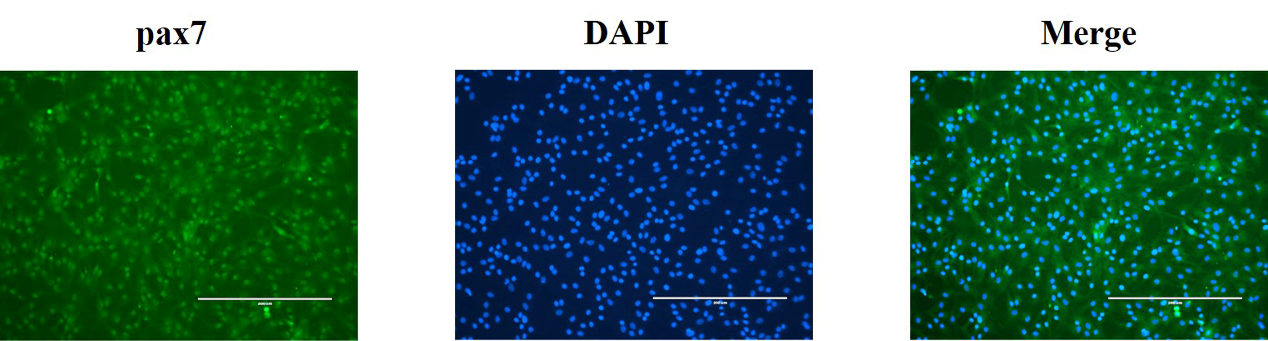
**

Fig.S1 Identification of isolated porcine skeletal muscle satellite cells
